# Analysis of Preplate Splitting and Early Cortical Development Illuminates the Biology of Neurological Disease

**DOI:** 10.3389/fped.2014.00121

**Published:** 2014-11-11

**Authors:** Eric C. Olson

**Affiliations:** ^1^Department of Neuroscience and Physiology, State University of New York Upstate Medical University, Syracuse, NY, USA; ^2^Developmental Exposure Alcohol Research Center (DEARC), Binghamton University, Binghamton, NY, USA

**Keywords:** preplate, reelin, fetal alcohol spectrum disorders, dendritogenesis, Golgi apparatus

## Abstract

The development of the layered cerebral cortex starts with a process called preplate splitting. Preplate splitting involves the establishment of prospective cortical layer 6 (L6) neurons within a plexus of pioneer neurons called the preplate. The forming layer 6 splits the preplate into a superficial layer of pioneer neurons called the marginal zone and a deeper layer of pioneer neurons called the subplate. Disruptions of this early developmental event by toxin exposure or mutation are associated with neurological disease including severe intellectual disability. This review explores recent findings that reveal the dynamism of gene expression and morphological differentiation during this early developmental period. Over 1000 genes show expression increases of ≥2-fold during this period in differentiating mouse L6 neurons. Surprisingly, 88% of previously identified non-syndromic intellectual-disability (NS-ID) genes are expressed at this time and show an average expression increase of 1.6-fold in these differentiating L6 neurons. This changing genetic program must, in part, support the dramatic cellular reorganizations that occur during preplate splitting. While different models have been proposed for the formation of a layer of L6 cortical neurons within the preplate, original histological studies and more recent work exploiting transgenic mice suggest that the process is largely driven by the coordinated polarization and coalescence of L6 neurons rather than by cellular translocation or migration. The observation that genes associated with forms of NS-ID are expressed during very early cortical development raises the possibility of studying the relevant biological events at a time point when the cortex is small, contains relatively few cell types, and few functional circuits. This review then outlines how explant models may prove particularly useful in studying the consequence of toxin and mutation on the etiology of some forms of NS-ID.

## Introduction

Neocortical development in human is initiated in the seventh week of gestation by the appearance of a layer of pioneer neurons, called the preplate or primordial plexiform layer (1, 2). Preplate neurons lie underneath the meninges and ultimately these neurons cover both cerebral vesicles. At this time, the human cortical wall is only ~250 μm thick, the majority of which is ventricular zone (VZ) (neural precursor cells) and the remaining 20–30 μm contains these early differentiating neurons of the preplate (3, 4). For comparison, the mouse preplate stage corresponds to embryonic day 12.5 post conception (E12.5), a time when the cortical wall has a similar composition of cells and a similar thickness (~150 μm) to the human preplate stage cortex.

Starting at the seventh to eighth week of gestation in human or E13.5 in mouse, a process called preplate splitting initiates the formation of cortical layering. In the mouse, preplate splitting begins in the lateral neocortex and proceeds dorsally and caudally over the next embryonic day (5). Preplate splitting is an early event in cortical development and involves the establishment of an organized layer of cortical plate (CP) neurons within the preplate. The establishment of future layer 6 (L6) neurons splits the preplate into a superficial layer of pioneer neurons called the marginal zone (MZ) and a deeper layer of pioneer neurons called the subplate (SP) (6–10). Preplate splitting is the first step in the formation of the layered cortex and is followed by the successive migration and lamination of cortical layers 5–2 in an inside out fashion (11).

## Pioneer Neurons of the Preplate

Both MZ neurons and SP neurons have essential roles in organizing the developing cortex (12, 13). MZ neurons, primarily Cajal–Retzius cells, secrete a critical chemotropic factor called Reelin (14) (discussed below) that is required for correct positioning of migrating CP neurons. SP neurons constitute a diverse group of cells (15, 16), which are essential for correct thalamocortical afferent targeting (17–19). Absent correct preplate splitting, the SP cells remain superficial in the cortex and both cortical layering (20) and thalamocortical targeting is disrupted (21, 22). Thus, preplate splitting is a fundamental event that enables the later assembly of the upper cortical layers and leads to a properly formed cerebral cortex (23–25).

## Gene Expression during Early Cortical Development

The significance of preplate splitting is underscored by the large number of genes specifically upregulated during this period. A prior study used fluorescence activated cell sorting to purify genetically labeled, developing L6 neurons (26). Sorted cells from the transgenic Eomes:eGFP^1^ mouse embryos (27) were subjected to RNA extraction and Affymetrix gene chip analysis. In these embryos, enhanced green fluorescent protein (eGFP) expression is under the control of the Eomes (Tbr2) promoter. Eomes is a transcription factor that is selectively expressed by intermediate neural precursor cells of the glutamatergic cortical lineage (28, 29). In transgenic embryos, GFP expression is transient but persists for several days in immature post mitotic neurons of the excitatory cortical lineage. By comparing the GFP^+^ population, primarily immature neurons, to the GFP-population, primarily neural precursors, up and down regulated genes in the differentiating excitatory cortical lineage were identified. Approximately half the genome was expressed by these neurons and more than 1000 genes show expression increases ≥2-fold during the first ~24–36 h after cell cycle exit (26). Genes of interest could then be validated by comparison to the Genepaint *in situ* database^2^. This prior study validated, and grouped by spatial expression pattern, 317 genes that were upregulated ≥3-fold during early cortical neuron differentiation. Importantly, over half of these highly upregulated genes have been associated with neuronal disease (26).

This dataset is a valuable resource that can be queried for genes specifically linked to neurological disorders including non-syndromic intellectual disability (NS-ID) (30). Of 46 human NS-ID genes identified previously (30), 43 were represented within this dataset, i.e., represented on the mouse Affymetrix Gene 1.0 ST Array (Table 1). Of these 43, 38 are expressed above a stringent threshold of RMA = 7.0, and these genes display an average expression level of RMA = 9.5, placing them in the approximate top third of all expressed genes in these immature neurons. At the onset of preplate splitting (E13.5), the expressed genes display an average increased expression of 1.6-fold in GFP^+^ neurons versus GFP-precursors. Surprisingly, only 2 genes of the 38 (MAGT and ARX) were downregulated ≥1.5-fold in differentiating neurons (i.e., more highly expressed in neural precursors than in differentiating neurons). Thus, the majority of identified NS-ID genes are highly expressed and upregulated by differentiating CP neurons during this early differentiation period, well prior to synapse formation.

**Table 1:** **List of non-syndromic intellectual disability (NS-ID) genes expressed in immature excitatory neurons**.

Gene	Affymetrix ID	Non-syndromic/syndromic	Gene name	Gene function	Protein localization	E13.5 GFP^−^ precursor RMA	E13.5 GFP^+^ neuron RMA	E14.5 GFP^+^ neuron RMA	E13.5 RMA fold-up	E14.5 RMA fold-up
ACSL4	10607089	NS	Acyl-CoA synthetase long-chain family member 4	Fatty acid metabolism	Mito	8.3	8.5	8.0	1.2	0.8
AFF2/FMR2	10599927	NS	Fragile X mental retardation 2	DNA binding protein/activator of transcription?	Nuc	8.0	9.9	9.7	3.7	3.2
AGTR2	10599001	NS/S	Angiotensin II receptor, type 2	G-protein-coupled receptor/programed cell death	PM	5.4	5.3	5.5	0.9	1.0
AP1S2	10603051	NS/S	Adaptor-related protein complex 1 sigma 2 subunit	Clathrin recruitment and sorting/synaptic vesicles	Golgi	8.8	9.3	9.2	1.4	1.3
ARHGEF6	10604713	NS	Rac/Cdc42 guanine nucleotide exchange factor 6	GEF for Rac and Cdc42	Cyto	6.1	4.5	4.7	0.3	0.4
ARX	10600755	NS/S	Aristaless related homeobox	Transcriptional regulation during development	Nuc	9.8	7.2	7.4	0.2	0.2
ATRX	10606263	NS/S	Transcriptional regulator ATRX	Chromatin remodeling	Nuc	10.2	10.5	10.4	1.2	1.2
BRWD3	10606393	NS/S	Bromo domain and WD repeat protein 3	JAK/STAT signaling in drosophila/chromatin modifier?	Nuc	8.7	9.3	9.0	1.5	1.2
CASK	10603708	NS/S	Calcium/calmodulin-dependent serine kinase	Kinase and scaffolding at synapses/MAGUK family protein	Syn, PM, Nuc, Cyto	10.3	11.5	11.4	2.2	2.2
CC2D1A	10580100	NS	Coiled-coil and C2 domain containing 1A	Transcriptional regulator/NF-κB pathway activator	Nuc, Cyto	7.6	7.9	7.9	1.2	1.2
CDH15	10576175	NS/S	Cadherin 15	Intercellular adhesion protein	PM	7.1	7.0	7.1	0.9	1.0
CRBN	10546775	NS	Cereblon	Expression of potassium channels	PM, Cyto	9.4	10.0	9.8	1.5	1.3
DLG3	10601062	NS	Synapse-associated protein 102	Post-synaptic density scaffold/MAGUK family protein	Syn, PM, ER, Cyto	8.9	9.5	9.5	1.5	1.5
DOCK8	10462140		Dedicator of cytokinesis 8	GEF?/F-actin organization	PM, Cyto, Nuc	6.1	6.1	6.1	1.0	1.0
FGD1	10602401	NS/S	Faciogenital dysplasia protein	GEF for Cdc42	Cyto	9.3	9.1	9.2	0.9	0.9
FTSJ1	10603508	NS	FtsJ homolog 1	rRNA processing	Nuc	9.8	9.9	9.7	1.1	0.9
GDI1	10600390	NS	GDP dissociation inhibitor 1	Inhibitor of Rab GTPases	Cyto	11.7	12.8	12.5	2.2	1.8
GRIK2	10368999	NS	Glutamate receptor, ionotropic„ kainate 2	Subunit of glutamate receptor (kainate)	PM, Syn	9.7	10.2	9.2	1.4	0.7
HUWE1	10602501	NS/S	HECT, UBA, and WWE domain containing 1	Ubiquitin E3 ligase/protein ubiquitination	Nuc, Cyto	10.5	10.5	10.6	1.0	1.0
IL1RAPL1	**NA**	NS	Interleukin 1 receptor accessory protein-like 1	Vesicle release/dendrite differentiation	PM	**NA**				
JARID1C/KDM5C	10602644	NS	Jumonji, AT rich interactive domain 1C	Transcriptional regulation/chromatin remodeling	Nuc	9.9	9.8	10.0	0.9	1.1
KIRREL3	10584165	NS/S	Kin of IRRE like 3	Synaptogenesis?	PM, Cyto, EC	7.5	8.9	9.3	2.5	3.3
MAGT1	10606301	NS	Magnesium transporter 1	Mg^2+^ uptake/N-glycosylation	ER	10.4	8.5	8.2	0.3	0.2
MBD5	10471967	NS/S	Methyl-CpG binding domain protein 5	Transcriptional regulation?	Nuc	7.8	8.5	8.5	1.6	1.6
MECP2	10605247	NS/S	Methyl-CpG binding protein 2	Transcriptional regulation	Nuc	8.8	9.0	9.1	1.1	1.2
NLGN4X	10601152	NS	X-linked neuroligin 4	Synaptic adhesion protein	PM, Syn	9.5	9.9	9.8	1.3	1.2
OPHN1	10605884	NS/S	Oligophrenin 1	Rho-GTPase activating protein	Cyto	8.2	9.3	9.3	2.2	2.1
PAK3	10602198	NS	p21-activated kinase 3	Effector of Rho-GTPases	Cyto	9.9	10.1	9.9	1.1	1.0
PQBP1	10603373	NS/S	Polyglutamine binding protein 1	Transcriptional regulation	Nuc, Cyto	10.9	11.3	10.8	1.3	1.0
PRSS12	10495854	NS	Neurotrypsin	Synaptic protease/cleaves agrin/synaptic plasticity	EC	6.9	7.3	7.1	1.3	1.2
PTCHD1	10607486	NS	Patched domain 1	Hedgehog receptor?	PM	6.4	6.5	6.4	1.1	0.9
RPS6KA3	10602772	NS/S	Ribosomal protein S6 kinase, 90kDa, polypeptide 3	Ras/Map/ERK regulation	Cyto	9.8	9.8	9.6	1.0	0.9
SHANK2	10559343	NS	SH3 and multiple ankyrin repeat domains 2	Scaffolding and cell adhesion protein/synaptic plasticity	Cyto, Syn	7.1	7.7	7.9	1.5	1.7
SHROOM4	10598240	NS/S	Shroom family member 4	Cytoskeletal architecture	Cyto	6.2	5.7	5.8	0.7	0.8
SLC6A8	10600210	NS	Solute carrier family 6 member 8	Creatine transporter	PM	8.4	8.3	8.5	0.9	1.1
STXBP1	10481711	NS	Syntaxin-binding protein 1	Synaptic vesicle docking and fusion/neurotransmission	PM, Syn, Cyto	8.6	10.8	10.9	4.6	5.0
SYNGAP1	10443091	NS	Synaptic Ras GTPase activating protein 1	NMDA receptor complex/Ras/Map/ERK regulation	PM, Syn	9.6	10.2	10.7	1.6	2.1
SYP	10598359	NS/S	Synaptophysin	Synaptic vesicle protein	Syn	8.8	10.9	10.6	4.2	3.5
TSPAN7	10598626	NS/S	Tetraspanin 7	Synapse maturation?	PM, Syn	11.9	11.7	11.2	0.8	0.6
TRAPPC9	**NA**	NS	NIK- and IKKB-binding protein	Neuronal NF-κB signaling/vesicular transport	Golgi, ER, Cyto	**NA**				
TUSC3	10571371	NS	Tumor suppressor candidate 3	Mg^2+^ uptake/oligosaccharide transferase/N-glycosylation	ER	9.3	10.2	10.2	1.8	1.8
UPF3B	10604078	NS/S	UPF3 regulator of nonsense transcripts homolog B	mRNA nuclear export and surveillance	Nuc, Cyto	7.9	8.1	8.0	1.1	1.1
ZNF41/zfp27 56%	**NA**	NS	Zinc finger protein 41	Putative repressor of transcription	Nuc	**NA**				
ZNF674/zfp182 56%	10603881	NS	Zinc finger protein 674	Putative repressor of transcription	Nuc	6.9	7.5	7.2	1.5	1.2
ZNF711/zfp711 98%	10601492	NS	Zinc finger protein 711	Activator of transcription?	Nuc	8.6	9.7	9.4	2.1	1.7
ZNF81/zfp160 47.4%	10442172	NS	Zinc finger protein 81	Repressor of transcription?	Nuc	8.3	8.9	8.6	1.5	1.2

*A dataset of genes expressed by immature mouse cortical neurons at E13.5 and E14.5 (26) was queried for the expression of human NS-ID orthologs identified in (30). The expression values are reported as RMA (robust multichip average) as a log2 scale (e.g., RMA 9.0 is twofold higher than RMA 8.0). The fold-up values are derived from comparing the expression of the gene in the GFP^+^ neuronal population to the expression of the gene in GFP-neural precursors. Highlighted rows identify genes that are either not represented in the mouse data set (NA) or are expressed at levels below threshold (RMA = 7.0). Mouse zinc finger protein (Zfp) orthologs are listed with their percent amino acid identity to the corresponding human zinc finger protein (ZNF). The human NS-ID table is modified from Kaufman et al. (30) with permission*.

*EC, extracellular; ER, endoplasmic reticulum; Golgi, Golgi apparatus; Syn, synapse; PM, plasma membrane; Nuc, nucleus; Mito, mitochondria*.

What functions might these NS-ID genes be performing during this early period? Expression analysis identified 15 out of 38 (40%) of these early expressed NS-ID gene products as being localized to the nucleus with most of these genes having functions in transcription, chromosomal remodeling, or RNA transport (Table 1) (30). An additional 10 of 38 (26%) of the predicted gene products localize to the plasma membrane, where they perform diverse functions as synaptic proteins, adhesion proteins, transporters, and receptors. This group includes Cadherin15 (CDH15) and a subunit of an ionotropic glutamate receptor (GRIK2). Four out of five NS-ID gene products that localize to the cytoplasm are involved in the regulation of small GTPases, namely, Rho, Cdc42, and Rab1, which coordinate cytoskeletal remodeling and vesicular transport, respectively. The five most upregulated NS-ID genes (STXBP1, SYP, FMR2, KIRREL3, and CASK) encode proteins with likely synaptic function. This was a surprise, since there are no morphologically identified synapses on L6 neurons at this time in development. While some of these early expressed mRNAs may not encode functional proteins, it is likely that many NS-ID genes have important roles during this very early period of cortical development.

## Cellular Dynamics during Early Cortical Development

This dynamic transcriptional profile may underlie the coincident processes of cortical neuron migration and molecular differentiation. Before achieving their mature form, cortical neurons are known to transition through multiple morphological states: from multipolar neuron to radial glial-associated migrating neuron to post migratory differentiating neuron (31–34). Immediately after cell cycle exit, the immature neuron adopts a multipolar morphology and migrates slowly through intermediate zone (IZ) (33, 34) while simultaneously initiating an axon (35, 36). The multipolar neuron, trailing an axon, continues migration until it reaches the SP, the layer of pioneer neurons that underlie the forming CP. At the SP, migrating neurons change from the multipolar shape to a bipolar shape coincident with their attachment to a radial glial fiber (37). The neuron, now apposed to the radial glial fiber, migrates through the developing CP in a saltatory (stepwise) fashion (38). As the neuron approaches the top of the CP, the neuron detaches from the radial glial fiber and translocates into position underneath the MZ (future layer 1) (38) where it elaborates an apical dendrite and becomes excitable.

**Figure 1:**
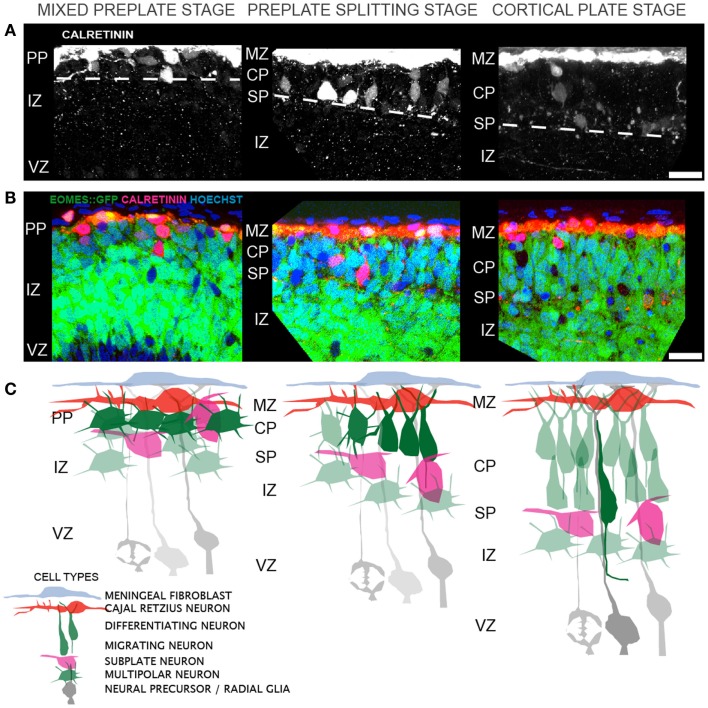
**Cellular events that contribute to cortical layer 6 formation and preplate splitting**. **(A)** Distribution of Calretinin immunopositive preplate neurons prior to (left), during (middle), and after (right) preplate splitting. **(B)** A single confocal slice through the Eomes:eGFP cortex at the same regions as in (A), showing the distribution of GFP^+^ excitatory cortical neurons (green) and Calretinin^+^ preplate neurons (red) and Hoechst^+^ nuclei (blue). **(C)** Model of the corresponding cellular rearrangements during the mixed preplate stage, where L6 neurons are intermingled with preplate neurons, the preplate splitting stage, where L6 neurons become radially oriented and coalesce into a recognizable cellular layer and the cortical plate stage, where migrating neurons enter the CP on radial glial fibers. Abbreviations: marginal zone (MZ), preplate (PP), cortical plate (CP), subplate (SP), intermediate zone (IZ), ventricular zone (VZ). Scale bar is 25 μm in **(A,B)**.

The sequence of morphological changes is less understood during the earlier period of preplate splitting. Two models have been proposed to account for the appearance of L6 neurons within the preplate. The first model posits direct somal translocation of the immature neuron from the VZ into the preplate (38, 39). This translocation involves the rapid movement of the nucleus into the leading process of the neuron and is thought to occur independent of radial glial guidance or attachment (38). Thus, as more L6 neurons translocate into the preplate, the preplate is split into the MZ and SP. This model, however, appears inconsistent with prior histological observations using electron microscopy (7, 40) and the Golgi stain method (7, 40), or with more recent observations made from the Eomes:eGFP embryonic cortex (10). In these animals, GFP expressing neurons of the excitatory lineage were found intermixed with Calretinin expressing preplate neurons, *prior* to preplate splitting (Figures 1A–C). Furthermore, below this mixture of preplate and L6 neurons lies a thick IZ composed of multipolar neurons that do not show translocating morphology (i.e., highly elongated in the radial direction). Thus, the GFP^+^ cells that are poised to enter the developing CP are not translocating from the VZ. Instead, preplate splitting appears to be initiated by polarized dendritic growth of L6 neurons and the concurrent coalescing of these L6 neurons into an organized and recognizable CP. The calcium-binding protein Calretinin is a marker for subsets of both MZ and SP neurons during early rodent cortical development (41, 42) and therefore the separation of preplate Calretinin^+^ cells into the MZ and SP groups is a hallmark of preplate splitting. In this model, Calretinin^+^ MZ neurons stay in place and Calretinin^+^ SP neurons either actively migrate away (43) from or are passively displaced by the coalescing L6 neurons. Thus, the initial phase of preplate splitting is driven by active reorganization of these L6 neurons, rather than their translocation. Future imaging studies should help resolve these two models. In both models, however, the period of preplate splitting represents a period of dynamic cellular transformations.

## Disruptions of Early Cortical Development

Disruptions of preplate splitting either by toxin or mutation (44) are associated with serious neurological disability including mental retardation, epilepsy (45), and possibly autism (46). Prenatal exposure to alcohol is a leading cause of mental retardation and intellectual disability (47, 48). The CDC estimates that 0.2–1.5 per 1000 live births are children with fetal alcohol syndrome (FAS), a syndrome defined by mental dysfunction (49). The cognitive deficits caused by prenatal exposure to EtOH are likely reflected in the specific functional and structural abnormalities found in brains of alcohol-exposed children (50, 51).

EtOH exposure is known to impact neuronal plasticity and these disruptions range from the short term (e.g., memory deficits caused by binge drinking) (52–54) to long term [e.g., disruption in memory and cognition associated with alcoholism (55)] to permanent [e.g., structural changes and intellectual disability associated with FASD (56, 57)] or chronic alcoholism (58). The disruptions caused by ethanol exposure vary with time period of exposure (59, 60). This differential sensitivity to ethanol may reflect the major underlying cellular processes occurring at the time of exposure (61).

Although EtOH exposure strongly promotes apoptosis during the synaptic formation period (62), EtOH can also target multiple events prior to synapse formation including neurogenesis, neuronal migration (63), axonal outgrowth (64, 65), and dendritic development (66–68). These biologically important processes can be assayed using early embryonic cortical explants. At this time, the cortex is small, composed of relatively few cell types and have few synapses. Nevertheless, these explants captures critical organotypic interactions including signals derived from other neurons as well as non-neuronal elements including radial glia (69, 70), blood vessels (71), meninges (72), and associated extracellular matrix (14). This organotypic environment provides the multiple substrates and signals that allow cortical neurons to mature through intermediate stages and to finally adopt appropriate form and function. Understanding how EtOH disrupts these signaling systems may be required for a fuller picture of the etiology FASD and the development of NS-ID.

## Whole Hemisphere Explants

A whole hemisphere explant procedure that permits 2 days of organotypic growth and encompasses the period of preplate splitting has been valuable in understanding the cellular transformations of preplate splitting (10, 73). In this procedure, entire embryonic cortices are isolated with the meninges intact and are then cultured on collagen filters as is done with slice explants (74, 75). Keeping the meninges intact helps preserve the organization of the basal lamina, the radial glial endfeet as well as the pioneer neurons that are found in the MZ. Disruptions of the meninges during development, through mutation (76, 77) or injury (78) can cause focal heterotopia and disrupt underlying cortical layering. Therefore, keeping the meninges as intact as possible is desirable and allows for continuous growth and lamination of the CP during the *in vitro* period. The CP is organized and shows appropriate expression of the transcription factors Tbr1 and Ctip2. Similarly, the radial glial network is intact evidenced by appropriate expression of the intermediate filament protein Nestin (73).

Using the whole hemisphere explant model, it was found that cellular orientation and apical dendritic growth was disrupted by single dose ethanol exposure, with an increase in primary dendrite number detected within 4 h of exposure (67). This dendritic alteration was accompanied by a morphological compaction of the Golgi apparatus, a key support organelle for the growing dendrite (79), as well as a slower reduction in cytoskeletal F-actin and the microtubule associated protein MAP2 content (67). These disruptions are remarkably similar to, but less severe than, disruptions caused by disruption of the Reelin-signaling pathway (80). Reelin is a large glycoprotein that is secreted by Cajal–Retzius cells in the MZ, during the period of preplate splitting (14, 81). Without Reelin, the preplate fails to split (9, 74, 75) and the subsequently generated cortical layers pile up underneath L6 leading to an inversion of cortical layering (20). In human beings, Reelin deficiency leads to mild epilepsy and severe mental retardation (45). At the cellular level in Reelin-deficient (*reeler*) cortical explants, neurons were tangentially oriented rather than radially oriented. The dendritic arbor was simplified and these neurons displayed more primary processes and a compact Golgi apparatus (10, 80). In addition dendritic expression of F-actin and MAP2 was reduced in *reeler* mutants compared to wild-type controls (10). Thus, studies using early cortical explants are showing a potential convergence of cellular phenotypes underlying two etiologically distinct forms of intellectual disability.

## Conclusion

Early cortical development is a period of remarkable dynamism with large scale changes in the pattern of gene expression, significant tissue growth and a surprising amount of neuronal differentiation. Disruption of early cortical development by exposure to toxin (e.g., EtOH) or mutation (e.g., Reln) can lead to intellectual disability. Explant models of early cortical development provide a bridge between dissociated culture studies and *in vivo* studies. The relatively small size and simple cellular composition allow for the study of disease relevant biology in the absence of synapses and functional circuits.

## Conflict of Interest Statement

The author declares that the research was conducted in the absence of any commercial or financial relationships that could be construed as a potential conflict of interest.

## Footnotes

^1^http://www.gensat.org

^2^http://www.genepaint.org
